# Tied Houses in Surgical Practice

**Published:** 1920-05-15

**Authors:** 


**The Editor's Letter Box—(*continued*).**

### Tied Houses in Surgical Practice.

*To the Editor of* The Hospital.

Sir,—If the article of your correspondent entitled "Tied Houses in Surgical Practice" represented a widespread and general condition in the profession (which I do not for a moment believe) it would indeed indicate a disgraceful state of affairs, and his condemnation of such a practice would surely be lamentably mild.

Dichotomy is an attempt to induce the operating surgeon to bribe the general practitioner to send cases to him. The patient is neither considered nor benefited thereby, and, even if no larger fee is demanded than would otherwise be asked, he stands to lose by being deprived of his right to exercise his choice in the selection of the consultant or operator, and still more in being unable to trust his medical adviser to recommend the best available consultant or operator in each case as the emergency arises.

Your correspondent compares dichotomy with the profits of a retail tradesman, and, with regard to the former, thinks that "as concerns ethics the crux of the matter" lies in the fact that "the public is unaware of the transaction." That, as he admits, is enough to condemn it, but a still more important consideration is that in dichotomy no service is rendered to the patient in return for the portion of the operation-fee pocketed by the general practitioner. There is no "good consideration," as there is in the case of the retail trader. Trade is good, but it is a dangerous thing to introduce trade into a profession.

What "legitimate boundaries" could there be to a system which would tend to put the pecuniary interests of the general practitioner in competition with, or even above, the real interests of his patient?

The service of the public and the good of the patient must be the essential considerations of every member of such an honourable profession as that of medicine—which includes surgery.—I am, yours truly,

A Retired Consulting Surgeon.

April 28, 1920.

